# Investigation of chikungunya arbovirus in cities of Bushehr province: An ELISA-based cross-sectional study

**DOI:** 10.1016/j.virusres.2026.199702

**Published:** 2026-02-17

**Authors:** Ebrahim Abbasi

**Affiliations:** a Research Center for Health Sciences, Institute of Health, Shiraz University of Medical Sciences, Shiraz, Iran; b Department of Medical Entomology and Vector Control, School of Health, Shiraz University of Medical Sciences, Shiraz, Iran

**Keywords:** Chikungunya virus, ELISA, Seroprevalence, Aedes mosquitoes, Bushehr province, Iran, Public health surveillance

## Abstract

**Background:**

Chikungunya virus (CHIKV) is a mosquito-borne alphavirus transmitted by *Aedes aegypti* and *Aedes albopictus*. Despite favorable ecological conditions in southern Iran, data on CHIKV exposure in Bushehr Province are lacking. This study aimed to determine the seroprevalence of CHIKV-specific IgG antibodies and assess potential viral circulation.

**Methods:**

A cross-sectional seroepidemiological survey was conducted from July to September 2023 in ten cities across Bushehr Province. Serum samples (n = 180) collected from volunteers at hospitals and blood donation centers were tested for CHIKV-specific IgG antibodies using a commercial ELISA kit.

**Results:**

Of the 180 serum samples analyzed, five tested positive for CHIKV IgG antibodies, yielding an overall seroprevalence rate of 2.78%. Seropositive individuals were identified in Bushehr, Dashtestan, and Dashti cities, indicating localized and possibly silent transmission.

**Conclusions:**

This study provides the first serological evidence of CHIKV exposure in Bushehr Province. The findings underscore the potential for local transmission in areas where competent *Aedes* vectors are present and highlight the urgent need for sustained vector surveillance, early warning systems, and public health preparedness in southern Iran.

## Highlights

• Investigated the Prevalence of the Chikungunya Virus in Bushehr Province, Iran, using the ELISA method.
• Out of 180 blood samples analyzed, 2.78% tested positive for Chikungunya virus antibodies.
• Study identified low circulation of Chikungunya virus, highlighting the need for surveillance.
• Findings emphasize the importance of vector control and public health preparedness in the region.
• Provides baseline data for future studies on vector-borne diseases in non-endemic regions of Iran.

## 1 Introduction

Chikungunya virus (CHIKV) is an arthropod-borne virus belonging to the genus *Alphavirus* within the family *Togaviridae*. It is primarily transmitted to humans through the bites of infected *Aedes aegypti* and *Aedes albopictus* mosquito’s species that are also vectors of dengue and Zika viruses. CHIKV infection manifests as an acute febrile illness characterized by fever, rash, and debilitating polyarthralgia, which in some cases may persist for several months, severely affecting patients’ quality of life and productivity. Since its first isolation in Tanzania in 1952, the virus has demonstrated remarkable epidemic potential, with major outbreaks reported across Africa, Asia, the Indian Ocean islands, and the Americas. Globalization, increased international travel, and adaptive mutations in CHIKV strains have facilitated its spread to previously non-endemic, temperate regions, underscoring its importance as a re-emerging global health threat(Abbasi, 2025b; Azari-Hamidian, 2023; McSweegan et al., 2015).

In recent decades, CHIKV has expanded its epidemiological range to the Middle East, where several outbreaks and sporadic cases have been reported in Saudi Arabia, Yemen, Pakistan, and the United Arab Emirates. The regional spread of the virus coincides with favorable climatic conditions that sustain the proliferation of *Aedes* mosquitoes. In these arid and semi-arid ecosystems, intermittent rainfall, improper water storage, and urbanization have created abundant larval habitats. The high degree of human mobility between Middle Eastern countries further facilitates viral introduction and transmission. Southern Iran, especially the coastal provinces along the Persian Gulf such as Bushehr, Hormozgan, and Sistan-Baluchestan, shares ecological and socio-environmental features conducive to *Aedes* vector establishment, including warm temperatures, high humidity, dense human settlements, and rapid urban and industrial development(Abbasi, 2025k; Paz and Semenza, 2016; Weaver and Lecuit, 2015).

Although Iran has not yet documented any large-scale outbreaks of CHIKV, several investigations have provided serological and entomological evidence of arboviral circulation in the country’s southern and southeastern regions. Studies conducted in Hormozgan and Sistan-Baluchestan Provinces have revealed the presence of *Aedes albopictus* and *Aedes aegypti*, indicating potential for local CHIKV transmission. Serological analyses have also detected CHIKV antibodies in human populations residing in these areas, suggesting prior exposure. Similarly, field surveillance along the Persian Gulf coastline has confirmed the establishment of *Aedes* populations capable of transmitting both dengue and chikungunya viruses. These findings collectively imply that southern Iran possesses the ecological prerequisites for arboviral emergence, especially in the context of climatic change and expanding human activity(Abbasi, 2025c; Abbasi, 2025f; Bakhshi et al., 2023; Tavakoli et al., 2020).

Despite mounting regional evidence, there remains a distinct lack of sero-epidemiological data on human exposure to CHIKV within Bushehr Province. This province’s coastal location, industrial growth, and maritime connections make it a potential hotspot for arboviral introduction via infected travelers or imported mosquitoes. Furthermore, its proximity to endemic neighboring countries, combined with suitable environmental conditions for *Aedes* proliferation, heightens the risk of viral establishment. The absence of prior serological surveys in Bushehr represents a major gap in Iran’s national arboviral surveillance framework. Given the co-circulation of multiple arboviruses in the Middle East, including dengue and Zika viruses, evaluating the serological footprint of CHIKV is essential to differentiate past exposures and assess regional vulnerability(Abbasi, 2025h; Abbasi, 2025p; Bakhshi et al., 2023; Bakhshi et al., 2020; Seyed-Khorami et al., 2024; Tavakoli et al., 2020).

Recent reports from entomological surveys have strengthened the concern regarding arboviral emergence in coastal Iran. Both *Aedes aegypti* and *Aedes albopictus* have been documented in multiple southern provinces, with confirmed breeding populations in Hormozgan and probable expansion toward Bushehr. Climate models predict that rising temperature and humidity, coupled with extensive human movement and urban water storage practices, will further enhance vector establishment. Under these circumstances, even limited serological evidence of CHIKV exposure warrants proactive surveillance and targeted vector management. Early identification of seropositive individuals can act as an epidemiological signal of silent transmission or imported infections preceding local outbreaks(Abbasi, 2025g; Abbasi, 2025n; Seyed-Khorami et al., 2024).

The present study was therefore designed to investigate, for the first time, the seroprevalence of anti-CHIKV IgG antibodies among residents of ten cities in Bushehr Province, southwestern Iran. Employing an ELISA-based approach, it aimed to determine the extent of prior human exposure to CHIKV and to provide essential baseline data for regional risk assessment. By integrating epidemiological insight with local ecological context, this research contributes to early-warning surveillance of arboviral infections and supports the development of evidence-based vector control and public health strategies in the Persian Gulf region. The outcomes are expected to guide future molecular and entomological investigations and enhance preparedness for potential arboviral emergence in Iran’s coastal provinces(Abbasi, 2025i; Abbasi, 2025l; Abbasi and Moemenbellah-Fard, 2025; Abbasi et al., 2025c).

## 2 Materials and methods

This study employed a cross-sectional seroepidemiological design to assess prior exposure to CHIKV among residents of Bushehr Province, southwestern Iran. Between July and September 2023, blood samples were collected from voluntary adult participants (≥18 years) attending hospitals and blood donation centers in ten cities of the province (Asaluyeh, Kangan, Bushehr, Dashtestan, Dashti, Deylam, Deyr, Ganaveh, Jam, and Tangestan). Eligibility criteria included permanent residence in Bushehr Province, absence of acute febrile illness at the time of sampling, and no recent travel (within three months) to CHIKV-endemic regions. Table 1 summarizes the demographic characteristics of the study population, including city of residence, sex, and age-group composition (Table 2).

**Table 1:** Demographic characteristics of study participants (n = 180) by city, sex, and age group - Bushehr Province, Iran (2023).

City	No. of Participants	Male n (%)	Female n (%)	Mean Age (Years ± SD)	Age Range (Years)
Bushehr	47	28 (59.6)	19 (40.4)	37.4 ± 9.8	18–61
Dashtestan	45	26 (57.8)	19 (42.2)	38.9 ± 11.1	20–63
Dashti	28	16 (57.1)	12 (42.9)	39.2 ± 10.4	19–62
Asaluyeh	12	8 (66.7)	4 (33.3)	35.1 ± 8.5	21–50
Kangan	13	7 (53.8)	6 (46.2)	36.7 ± 9.1	22–56
Deylam	11	7 (63.6)	4 (36.4)	37.8 ± 10.2	18–60
Deyr	7	4 (57.1)	3 (42.9)	34.5 ± 9.3	20–53
Ganaveh	7	5 (71.4)	2 (28.6)	36.1 ± 9.8	24–59
Jam	5	3 (60.0)	2 (40.0)	39.6 ± 10.0	26–58
Tangestan	5	3 (60.0)	2 (40.0)	38.3 ± 8.7	27–54
Total	**180**	**107 (59.4)**	**73 (40.6)**	**38.2 ± 10.6**	**18–65**

**Caption**: The table summarizes the distribution of participants according to city of residence, sex, and age. Participants were residents of ten cities in Bushehr Province, with a higher proportion of males (59.4%) than females (40.6%). The mean age was 38.2 ± 10.6 years (range: 18–65 years).

**Table 2:** Eligibility Criteria for Study Participation.

Category	Criteria
Inclusion Criteria	Permanent resident of Bushehr Province; age ≥18 years; written informed consent; no acute febrile illness at sampling; no travel to arbovirus-endemic areas in previous 3 months
Exclusion Criteria	Refusal of consent; symptoms of active infection; recent travel to endemic regions; inadequate serum volume or quality; age <18 years

**Caption:** Summary of inclusion and exclusion criteria applied to participant recruitment in the CHIKV seroprevalence study.

Approximately 5 mL of peripheral venous blood was collected from each participant using standard sterile procedures. Serum was separated, aliquoted, and stored at −20°C until laboratory analysis. Detection of anti-CHIKV IgG antibodies was performed using a commercially available enzyme-linked immunosorbent assay (ELISA) kit (Euroimmun, Lübeck, Germany), following the manufacturer’s instructions. Samples were classified qualitatively as positive or negative based on optical density values relative to the manufacturer-defined cut-off (Table 3).

**Table 3:** Key Study Variables and Outcome Definitions.

Variable Category	Variable	Definition / Measurement
Primary Outcome	CHIKV IgG serostatus	Positive or negative based on ELISA OD values relative to manufacturer-defined cut-off
Demographic Variables	Age	Years (categorized for descriptive analysis)
	Sex	Male / Female
	City of residence	One of ten cities in Bushehr Province
Eligibility Variables	Travel history	No travel to endemic areas within 3 months
	Clinical status	No acute febrile illness at sampling
Laboratory Controls	Positive control	Manufacturer-supplied CHIKV IgG–positive serum
	Negative control	Manufacturer-supplied CHIKV IgG–negative serum

**Caption:** Summary of dependent and independent variables analyzed in the study.

Descriptive statistical analyses were conducted using SPSS software (version 26.0). Seroprevalence was calculated as the proportion of IgG-positive samples among the total number tested and summarized overall and by city. Due to the limited number of positive cases and the exploratory nature of the study, no inferential statistical tests were applied.

A detailed description of sample size calculation, laboratory procedures, assay validation, variable definitions, and extended statistical considerations is provided in **Supplementary Methods S1.** Additional methodological details, including sample size calculation, extended ELISA procedures, assay performance characteristics, and variable definitions, are provided in **Supplementary Methods S1**(Abbasi, 2025d; Abbasi, 2025f; Abbasi, 2025q; Abbasi, 2025s; Abbasi, 2026a; Abbasi, 2026b).

## 3 Results

A total of 180 serum samples were successfully collected from voluntary participants residing in ten cities of Bushehr Province, including Asaluyeh, Kangan, Bushehr, Dashtestan, Dashti, Deylam, Deyr, Ganaveh, Jam, and Tangestan. All specimens were tested using an ELISA assay for the detection of anti–CHIKV IgG antibodies. Among the analyzed samples, five tested positive, corresponding to an overall seroprevalence rate of 2.78%, while 175 samples (97.22%) were negative. The detection of CHIKV-specific antibodies, although at a low frequency, demonstrates evidence of prior human exposure to the virus within the study region. The presence of these seropositive individuals is particularly notable because Bushehr Province had not previously been investigated for CHIKV exposure, suggesting a possible introduction or limited silent circulation of the virus(Abbasi, 2025a; Abbasi, 2025e; Abbasi and Moemenbellah-Fard, 2025).

**Fig. 1:**
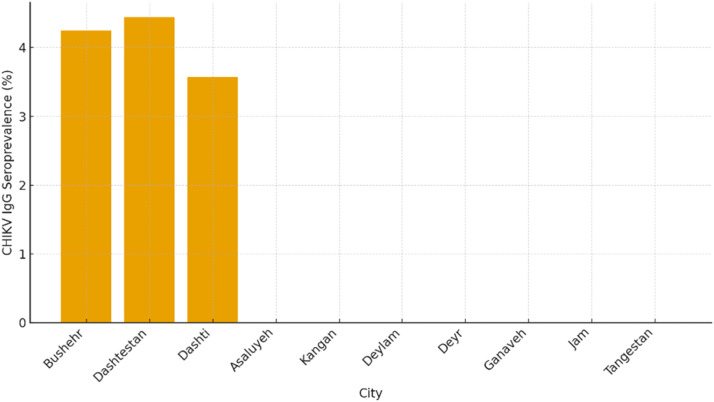
Distribution of CHIKV IgG seroprevalence (%) by city, Bushehr Province, Iran (2023). **Caption**: Bar chart showing the percentage of CHIKV IgG–positive serum samples among 180 participants from ten cities of Bushehr Province. Seropositive cases were detected only in Bushehr (4.25%), Dashtestan (4.44%), and Dashti (3.57%), indicating localized and low-level exposure to CHIKV. No positive samples were found in Asaluyeh, Kangan, Deylam, Deyr, Ganaveh, Jam, or Tangestan. The data illustrate a limited pattern of CHIKV seroprevalence, with detectable positivity confined mainly to the central and northern districts of the province.

**Fig. 2:**
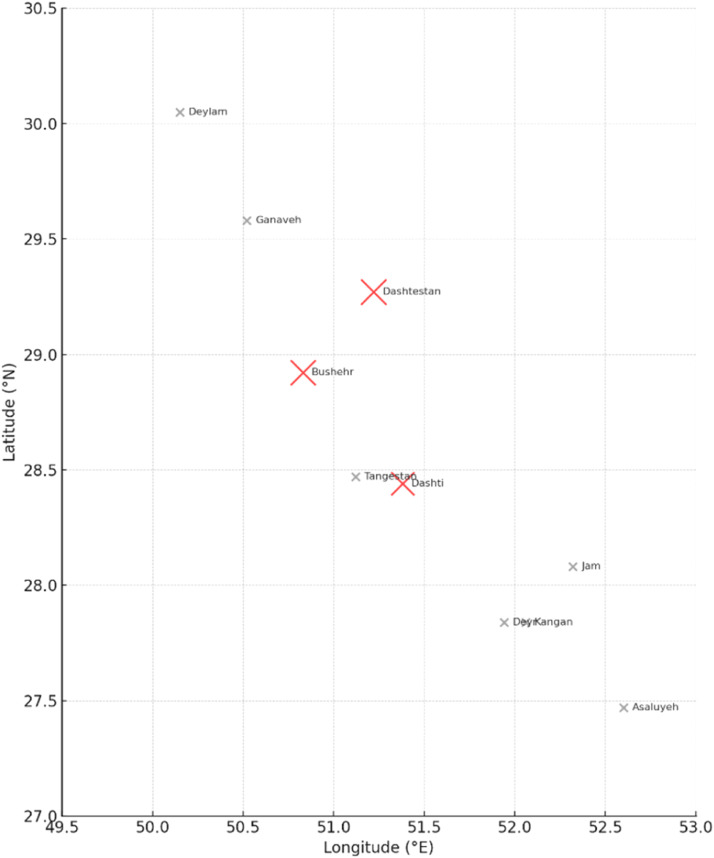
Geographic distribution of CHIKV IgG seroprevalence by city, Bushehr Province, Iran (2023). **Caption**: Map visualization showing the approximate geographic locations of the ten surveyed cities in Bushehr Province, with circle size proportional to the CHIKV IgG seroprevalence (%) observed in each city. Red circles represent cities with seropositive individuals Bushehr, Dashtestan, and Dashti located mainly in the central and northern parts of the province. Gray markers represent cities where no CHIKV-positive samples were detected. The geographic clustering of low-level seropositivity highlights potential zones for focused vector surveillance and public health interventions.

The geographic distribution of CHIKV IgG–positive cases showed a localized pattern restricted to three of the ten surveyed cities. Two positive samples were identified in Bushehr City (4.25%), two in Dashtestan (4.44%), and one in Dashti (3.57%). No seropositive samples were detected in Asaluyeh, Kangan, Deylam, Deyr, Ganaveh, Jam, or Tangestan. The city-wise distribution of CHIKV seroprevalence is summarized in Table 4, and the corresponding spatial representation is illustrated in Fig. 1. As depicted, the detected seropositivity was primarily confined to the central and northern parts of the province, where population density, domestic travel, and industrial activity are comparatively higher. This spatial clustering pattern suggests possible microfoci of exposure, potentially influenced by ecological or demographic factors favorable to *Aedes* mosquito proliferation. The mapped distribution of positive cases across Bushehr Province, visualized in Fig. 2, further supports the geographically limited yet epidemiologically significant nature of CHIKV exposure.

**Table 4:** Distribution of CHIKV IgG seroprevalence among study participants by city, age group, and sex, Bushehr Province, Iran (July–September 2023).

No.	City	Blood collection centers	Frequency	Number of Tests	Chikungunya (Positive Results)
1	Asaluyeh/ Kangan	Nabi Akram Hospital	20	8	3 (-)
		Kangan Blood Donation Center	150	61	20 (-)
2	Bushehr	Bushehr Blood Donation Center	150	61	19 (-), 1 (+)
		Amir al-Mominin Hospital	50	20	6 (-)
		Persian Gulf Martyrs Hospital	150	61	21 (-)
3	Dashtestan	Dashtestan Blood Donation Center	150	61	19 (-), 2 (+)
		17 Shahrivar Hospital Clinic	30	12	4 (-)
		Navid 24-hour Clinic	20	8	3 (-)
4	Dashti	Zainabiya Hospital	200	82	26 (-), 2 (+)
5	Deylam	Baghiullah Hospital	30	12	4 (-)
6	Deyr	Deyr Blood Donation Center	150	61	22 (-)
7	Ganaveh	Ganaveh Blood Donation Center	150	61	21 (-)
8	Jam	Tawheed Hospital	20	8	2 (-)
9	Tangestan	Imam Hossein Hospital	50	16	5 (-)
	Total		1320	532+8*	175 (-), 5 (+)

⁎ Note: The total number of tests includes additional tests not detailed in the table.

Demographic analysis of the study population indicated that out of the 180 participants, 107 (59.4%) were male and 73 (40.6%) were female, with ages ranging from 18 to 65 years and a mean age of 38.2 ± 10.6 years. Detailed demographic characteristics, including participant distribution by city, age group, and sex, are presented in Table 1. The occurrence of CHIKV IgG antibodies showed no apparent sex-based difference: three of the seropositive individuals (60%) were male, and two (40%) were female. Similarly, no statistically significant differences were observed between male and female participants regarding antibody detection. Analysis of age-specific distribution revealed that most seropositive individuals (three out of five, or 60%) belonged to the 31–45-year age group, while one seropositive case occurred in the 18–30-year group and another in the 46–60-year group. This pattern suggests that middle-aged adults potentially due to occupational exposure and outdoor activities may have a relatively higher likelihood of encountering infected mosquitoes(Abbasi, 2025m; Nyamwaya et al., 2021).

Overall, the low seroprevalence observed in this survey indicates that CHIKV circulation within the human population of Bushehr Province remains limited. Nonetheless, the detection of IgG-positive individuals demonstrates the potential for viral introduction and silent transmission, especially considering the confirmed presence of competent *Aedes* vectors in southern Iran. The spatial heterogeneity of positive results implies that localized environmental conditions, such as proximity to vector breeding habitats, urban density, and industrial development zones, may influence exposure risk. Fig. 1, Fig. 2 illustrate the comparative seroprevalence by city and the geographic distribution of antibody-positive cases across Bushehr Province. Taken together, these findings provide the first serological evidence of CHIKV exposure in Bushehr Province and underscore the importance of continuous arboviral surveillance(Abbasi, 2025j; Bakhshi et al., 2020; Soni et al., 2023).

Although the proportion of positive samples was low, their detection across multiple cities suggests that CHIKV may already exist in the region at a subclinical level. The absence of reported clinical cases during the sampling period further implies that infections could have been asymptomatic or misdiagnosed as other febrile illnesses such as dengue or influenza-like syndromes. This possibility emphasizes the necessity for molecular confirmation in future investigations to distinguish true CHIKV infections from serologically cross-reactive arboviruses. In this study, the ELISA-based detection of IgG provided reliable evidence of previous exposure, offering valuable baseline data for subsequent molecular and entomological research in the area(Abbasi, 2025l; Abbasi et al., 2025a; Pouriayevali et al., 2019).

## 4 Discussion

The present study provides the first serological evidence of exposure to the CHIKV among human populations in Bushehr Province, southwestern Iran. Although the overall seroprevalence rate was low (2.78%), the detection of CHIKV-specific IgG antibodies confirms that the virus has been introduced to the region, either through imported infections or silent, localized transmission. This finding expands the known geographical range of CHIKV exposure in Iran and aligns with the growing evidence of arboviral activity along the southern coastal provinces. The low but detectable antibody presence suggests a recent or limited introduction, reflecting the early phase of potential viral establishment(Abbasi et al., 2025b; Abbasi et al., 2025d).

The serological findings from Bushehr Province are consistent with earlier research conducted in other parts of Iran and neighboring Middle Eastern countries, where sporadic cases and low-level CHIKV circulation have been observed. Similar studies in Sistan-Baluchestan and Hormozgan Provinces reported comparable IgG seropositivity rates, indicating limited but persistent exposure among residents. These data collectively suggest that CHIKV is silently circulating in certain parts of southern Iran, supported by ecological conditions favorable to *Aedes* vectors. Comparable serological results have been documented across regions of the Arabian Peninsula, Pakistan, and East Africa, where climatic and demographic features mirror those of Bushehr. The convergence of these findings highlights the increasing vulnerability of the Persian Gulf region to arboviral incursions driven by globalization, climate variability, and mosquito adaptation(Abbasi, 2025r; Paz and Semenza, 2016; Pouriayevali et al., 2019; Semenza et al., 2016; Soni et al., 2023).

The limited seroprevalence observed in this study likely reflects the early stages of viral introduction or transient transmission. The absence of acute febrile illness among participants suggests that infections were either asymptomatic or misclassified as other febrile conditions such as dengue or influenza-like syndromes. This pattern of subclinical infection is consistent with the epidemiology of CHIKV in non-endemic regions, where population immunity remains low, and exposure events are sporadic. Although no confirmed autochthonous transmission has yet been reported in Iran, the detection of CHIKV antibodies among residents of Bushehr Province implies prior exposure within or near the region. Given the province’s active maritime trade and frequent population exchange with endemic countries, such as Pakistan and Yemen, imported infections may have played a critical role in viral introduction. The persistence of competent mosquito vectors, combined with increasing urbanization and climate-induced habitat suitability, could facilitate future localized transmission if not closely monitored(Abbasi, 2025o; Abedi-Astaneh et al., 2025; Chinikar et al., 2013; Pouriayevali et al., 2019).

Entomological surveillance studies conducted in recent years have reported the establishment of *Aedes aegypti* and *Aedes albopictus* populations in several southern provinces, including Hormozgan, Sistan-Baluchestan, and Bushehr. These findings provide a crucial ecological context for interpreting the serological evidence of CHIKV exposure. The presence of competent vectors within Bushehr Province, especially in densely populated and industrialized zones, enhances the risk of local transmission once viremic individuals are introduced. Environmental and anthropogenic factors, such as increasing temperature, humidity, urban expansion, and improper water storage, further contribute to the proliferation of *Aedes* mosquitoes. Therefore, the detection of even a small number of seropositive individuals should be regarded as an early warning signal necessitating integrated entomological, serological, and molecular surveillance program(Azari-Hamidian, 2023; Khoobdel et al., 2022; Seyed-Khorami et al., 2024; Shoushtari et al., 2022; Zadeh et al., 2023). From an epidemiological perspective, the circulation of togaviruses, particularly alphaviruses, in Iran appears to be highly limited. Among medically important alphaviruses, Chikungunya virus is the only species for which human exposure has been documented through serological and molecular evidence in the country. Other alphaviruses known to cause human disease elsewhere, including Ross River virus, Mayaro virus, O’nyong-nyong virus, and Sindbis virus, have not been reported to circulate endemically in Iran, nor have they been identified through routine surveillance or outbreak investigations. Consequently, the likelihood that detected IgG antibodies in the present study reflect cross-reactivity with non-CHIKV alphaviruses is considered low. This epidemiological context, combined with the high reported specificity of the commercial CHIKV IgG ELISA assay, supports the interpretation that seropositive results most likely indicate prior exposure to Chikungunya virus rather than infection with another alphavirus.

The low prevalence of CHIKV antibodies observed in this study is not unexpected for a region undergoing early arboviral emergence. This trend mirrors historical patterns seen in other geographic regions where CHIKV initially appeared at low frequencies before escalating into larger outbreaks once ecological and demographic thresholds were met. It is plausible that CHIKV circulation in Bushehr remains limited due to insufficient vector density or transient introductions that fail to establish sustained transmission cycles. However, the co-occurrence of other arboviruses, such as dengue, in southern Iran raises the possibility of overlapping transmission cycles facilitated by the same vector species. Such interactions may influence vector competence and viral persistence, thereby complicating outbreak prediction and control(Aghaie et al., 2014; Poudine et al., 2023; Pouriayevali et al., 2019).

From a methodological perspective, this study employed an ELISA-based serological approach to detect CHIKV IgG antibodies, which is well-suited for identifying prior infections and assessing cumulative exposure. However, it cannot distinguish between recent and remote infections or between closely related alphaviruses, given possible cross-reactivity. Future investigations should incorporate confirmatory tests such as plaque reduction neutralization tests (PRNT) or molecular diagnostics (RT-PCR) to validate antibody-based findings and assess active infections. Moreover, entomological monitoring and vector infection assays would be essential to confirm the presence of CHIKV within *Aedes* populations and clarify the virus’s transmission dynamics in the region. Integrating these approaches will provide a comprehensive understanding of CHIKV epidemiology and help delineate the relative contribution of imported versus locally acquired infections(Abbasi, 2025e; Abedi-Astaneh et al., 2025; Lam et al., 2001; Seyed-Khorami et al., 2024). Although the detection of anti-CHIKV IgG antibodies provides valuable evidence of prior exposure, it is important to acknowledge the inherent limitation of serological assays with respect to cross-reactivity among alphaviruses. While cross-reactivity cannot be completely excluded, its impact in the present study is likely minimal, as no other endemic or epidemic alphaviruses are known to circulate in Iran. Furthermore, the commercial ELISA assay used has been reported to exhibit high specificity for CHIKV, with limited cross-reactivity in independent evaluations. Nevertheless, the results should be interpreted as indicating *probable* past exposure to CHIKV rather than laboratory-confirmed infection. Future studies incorporating plaque reduction neutralization tests (PRNT) or molecular assays would be valuable to further confirm specificity and characterize viral circulation dynamics. The absence of confirmatory neutralization assays represents an important limitation of this study. Neutralization tests such as PRNT or FRNT are considered the reference standard for differentiating CHIKV antibodies from those directed against antigenically related alphaviruses. While the epidemiological context and high reported specificity of the ELISA assay support the interpretation of the detected antibodies as CHIKV-specific, definitive confirmation would require neutralization testing. Future investigations should prioritize PRNT or FRNT analysis of ELISA-positive samples, ideally in combination with molecular detection and entomological surveillance, to provide a more comprehensive assessment of CHIKV circulation in southern Iran. fNevertheless, the possibility of undetected or historically unrecognized alphavirus exposure cannot be completely excluded. Although the ELISA assay used in this study has high reported sensitivity and specificity, confirmatory neutralization assays such as plaque reduction neutralization tests (PRNT) were not performed, which should be considered in future studies to further exclude potential serological cross-reactivity. Because Chikungunya virus is not endemic in Iran, independently confirmed local CHIKV IgG–positive human sera were not available for use as external controls; therefore, assay validation relied on the internal positive and negative controls supplied with the commercial ELISA kit.

While the study’s cross-sectional design provides valuable baseline data, it is inherently limited in its ability to capture temporal trends in transmission. The sample size, though representative for an initial investigation, restricts statistical inference and spatial extrapolation. Additionally, the study focused solely on adults attending healthcare and blood donation centers, which may exclude populations with differing exposure risks, such as children or rural residents. Despite these constraints, the findings establish an important foundation for future longitudinal studies, molecular surveillance, and vector ecology research. These insights will be crucial for refining public health preparedness and developing predictive models for arboviral emergence in Iran’s coastal provinces(Abbasi and Moemenbellah-Fard, 2025). Although this study focused exclusively on CHIKV, the co-circulation of other arboviruses such as dengue virus in southern Iran underscores the need for integrated arboviral surveillance. While serological cross-reactivity can occur among closely related alphaviruses, such as o’nyong-nyong virus, it is not expected between alphaviruses and flaviviruses, supporting the specificity of the CHIKV IgG findings reported here.

## 5 Conclusion

This study provides the first serological evidence of probable prior exposure to CHIKV among residents of Bushehr Province, indicating that the virus may have been introduced into the region, likely through imported cases or low-level silent transmission. Although the overall seroprevalence rate of 2.78% is low, its detection across multiple cities suggests that the ecological and climatic conditions of the province are suitable for potential viral establishment. These findings highlight the need for continuous surveillance to prevent local transmission and mitigate future outbreaks. The results underscore the importance of strengthening arboviral monitoring systems in southern Iran through integrated approaches combining serological, molecular, and entomological surveillance. Given the widespread presence of competent *Aedes* vectors and the ongoing expansion of urban and industrial areas, there is an urgent need to implement vector control strategies, enhance diagnostic capacity, and promote public health education. Establishing an early warning network for vector-borne diseases, particularly in high-risk coastal regions, will be instrumental in minimizing the potential impact of emerging arboviruses such as CHIKV. Finally, the present findings contribute to the broader understanding of arboviral epidemiology in Iran by filling a critical geographical gap in national surveillance data. By demonstrating evidence of CHIKV exposure in Bushehr Province, this study provides a scientific foundation for future risk assessments, policymaking, and vector management programs. Continuous, multidisciplinary research integrating climate modeling, vector biology, and human serology will be essential to predict and control arboviral threats in the rapidly changing ecological landscape of Iran’s southern coastal provinces(Poudine et al., 2023; Seyed-Khorami et al., 2024).

## Ethical approval and consent to participate

This study was conducted in accordance with the Declaration of Helsinki and was approved by the Ethics Committee of Shiraz University of Medical Sciences (Approval ID: IR.SUMS.SCHEANUT.REC.1401.121). Written informed consent was obtained from all participants prior to blood sample collection.

## Ethical considerations

This study was approved by the Ethics Committee of Shiraz University of Medical Sciences (Ethics approval ID: IR.SUMS.SCHEANUT.REC.1401.121). All participants were informed about the purpose and procedures of the study, and written informed consent was obtained from each participant prior to blood sample collection. Participation was voluntary, and all procedures were conducted in accordance with the ethical standards of the institutional research committee and the Declaration of Helsinki.

## Funding

This research was supported by Shiraz University of Medical Sciences (SUMS) under proposal number 25843 as part of a doctoral dissertation project. The funding body had no role in the study design; data collection, analysis, or interpretation; manuscript preparation; or the decision to submit the article for publication.

## Transparency statement

The author affirms that this manuscript is an honest, accurate, and transparent account of the study being reported; that no important aspects of the study have been omitted; and that any deviations from the original study plan have been fully disclosed and explained.

## CRediT authorship contribution statement

**Ebrahim Abbasi:** Writing – review & editing, Writing – original draft, Visualization, Validation, Supervision, Software, Resources, Project administration, Methodology, Investigation, Funding acquisition, Formal analysis, Data curation, Conceptualization.

## Declaration of competing interest

The authors declare that they have no known competing financial interests or personal relationships that could have appeared to influence the work reported in this paper.

## Data Availability

All data generated or analyzed during this study are included in this published article.

## Appendix Supplementary materials

Image, application 1
