# Supplementary material for: Investigation of chikungunya arbovirus in cities of Bushehr province: An ELISA-based cross-sectional study
Source: Virus Res. 2026 Feb 17;366:199702. doi: 10.1016/j.virusres.2026.199702 (PMC12945641; doi:10.1016/j.virusres.2026.199702)
Supplement: Supplementary file 1 [file mmc1.docx]

***Materials and Methods***

1. ***Study Area and Population***

This study was conducted in Bushehr Province, located in the southwestern region of Iran, along the coast of the Persian Gulf. The province is characterized by a semi-arid to arid climate, with high temperatures and moderate humidity, conditions that are conducive to the breeding and survival of *Aedes* mosquito vectors. Given the province’s coastal geography, rapid urbanization, and increased human mobility due to industrial activities particularly in the oil and gas sectors the region is potentially vulnerable to the emergence and transmission of arboviral diseases such as Chikungunya. The target population for this seroepidemiological study consisted of residents from ten cities within the province, including Asaluyeh, Kangan, Bushehr, Dashtestan, Dashti, Deylam, Deyr, Ganaveh, Jam, and Tangestan. Blood samples were collected from voluntary participants attending blood donation centers and hospitals in each city. Individuals of various age groups and both sexes were included in the study, with the inclusion criteria requiring no recent travel history to endemic areas and no acute febrile illness at the time of sample collection. This diverse geographic and demographic coverage was intended to provide a comprehensive overview of the potential circulation of the Chikungunya virus in different urban and semi-urban contexts across the province. Detailed demographic and sampling information for all participants, including age, sex, travel history, and sampling source, are presented in Supplementary Table S1.

1. ***Inclusion Criteria and Exclusion Criteria***

**Inclusion Criteria:**

Participants were eligible for inclusion in the study if they met the following conditions:

1. They were permanent residents of one of the ten selected cities in Bushehr Province.
2. They provided written informed consent to participate in the study.
3. They were aged 18 years or older, regardless of sex.
4. They had no clinical symptoms of acute febrile illness at the time of blood sample collection.
5. They had no recent travel history (within the past three months) to regions endemic for Chikungunya virus or other arboviral infections.
6. They attended blood donation centers or hospitals for routine services unrelated to acute infectious diseases.

**Exclusion Criteria:**

Individuals were excluded from the study if they met any of the following criteria:

1. They declined to provide informed consent or withdrew their participation at any point during the study.
2. They exhibited signs or symptoms suggestive of an active infectious disease, such as fever, rash, or joint pain at the time of sampling.
3. They had traveled outside the province or to Chikungunya-endemic areas within the three months preceding the study.
4. Their blood samples were insufficient in volume or did not meet quality standards for ELISA testing.
5. They were under 18 years of age or otherwise unable to provide autonomous consent
6. ***Blood Sampling***

Blood sample collection was carried out at various designated healthcare facilities and blood donation centers across ten cities in Bushehr Province, including Asaluyeh, Kangan, Bushehr, Dashtestan, Dashti, Deylam, Deyr, Ganaveh, Jam, and Tangestan. All participants were informed about the purpose and procedures of the study, and written informed consent was obtained prior to sample collection. A standard venipuncture technique was employed to collect approximately 5 mL of peripheral venous blood from each participant under sterile conditions. The collected samples were transferred into sterile, labeled tubes and were subsequently centrifuged to separate serum. Serum samples were then aliquoted and stored at –20°C until laboratory analysis. To ensure the integrity and traceability of the specimens, each sample was assigned a unique identification code. Strict protocols were followed during handling, storage, and transportation to prevent sample degradation or contamination. The entire process adhered to biosafety and ethical guidelines approved by the Ethics Committee of Shiraz University of Medical Sciences (Approval ID: IR.SUMS.SCHEANUT.REC.1401.121). For each participant, demographic variables (age, sex, and city of residence), travel history (both within Iran and abroad), and the source of blood collection (hospital or blood donation center) were recorded using a standardized questionnaire. These data were used to interpret potential exposure history and to identify possible imported cases of CHIKV infection.

1. ***Sample Size Calculation***

The sample size for this cross-sectional seroepidemiological study was determined based on standard statistical principles for prevalence estimation in a finite population. Considering the lack of prior comprehensive data on the seroprevalence of *Chikungunya virus (CHIKV)* in Bushehr Province, a conservative expected prevalence of 5% was assumed to maximize sample size. The following formula for sample size estimation in prevalence studies was used:


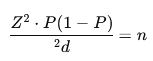


Where:

- *n* = required sample size
- *Z* = standard normal deviate corresponding to the desired confidence level (1.96 for 95% confidence)
- *P* = estimated prevalence (0.05)
- *d* = desired precision or margin of error (0.03)

Substituting these values:


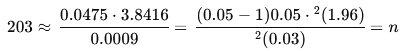


Taking into account potential sample loss, non-responses, or invalid samples, a total of 180 samples were ultimately collected due to practical limitations, including participant availability and resource constraints. Although slightly lower than the estimated ideal, this sample size still allows for meaningful inference regarding the presence and distribution of CHIKV antibodies in the study population, especially when interpreted in the context of early surveillance and exploratory research. Table 1 summarizes the demographic characteristics of the study population, including city of residence, sex, and age-group composition.

1. ***Sample Collection Period***

The blood sample collection was conducted over a three-month period, from July to September 2023, which corresponds to the hot and humid season in Bushehr Province. This period was intentionally selected due to the increased activity of *Aedes* mosquito vectors, which are known to proliferate under such climatic conditions, thereby heightening the potential risk for arboviral transmission, including *Chikungunya virus (CHIKV).* Coordinated fieldwork was carried out in collaboration with local blood donation centers and healthcare facilities across ten cities in the province. All procedures were standardized across sites to ensure consistency in sample handling, labeling, and storage. The timing of the sample collection also coincided with heightened public health surveillance efforts in response to increased regional awareness of vector-borne diseases, thereby facilitating participant recruitment and community engagement.

1. ***ELISA Testing***

The detection of anti-*Chikungunya virus (CHIKV)* IgG antibodies in human serum samples was carried out using the Enzyme-Linked Immunosorbent Assay (ELISA) technique, a widely accepted method for serological surveillance. The assay was performed using a commercially available enzyme-linked immunosorbent assay (ELISA) kit for the qualitative detection of anti-Chikungunya virus (CHIKV) IgG antibodies in human serum (ELISA Anti-Virus Chikungunya IgG; commercial manufacturer), and the test was not developed in-house. The specific diagnostic kit employed was the *ELISA Anti-Virus Chikungunya (IgG)* test, designed for in vitro qualitative detection of CHIKV-specific antibodies in human serum or plasma. All reagents and samples were first brought to room temperature before use. The ELISA microplate wells, pre-coated with CHIKV antigens, were used to capture specific antibodies present in the serum. A 100 µL volume of each test serum, together with manufacturer-supplied CHIKV IgG–positive and CHIKV IgG–negative control sera, was added to designated wells. Plates were incubated at 37°C for 1 hour, followed by a series of washing steps to remove unbound components. Each assay run included manufacturer-provided positive and negative control sera with known Chikungunya virus IgG reactivity. These controls were used to validate assay performance, confirm proper reagent function, and ensure accurate determination of the assay cut-off value for each plate. Subsequently, 100 µL of enzyme-conjugated anti-human IgG was added to each well, and the plates were incubated for an additional 30 minutes at 37°C. After another washing cycle, 100 µL of TMB (3,3',5,5'-tetramethylbenzidine) substrate solution was added, and the reaction was allowed to proceed for 10–15 minutes in the dark at room temperature. The enzymatic reaction was stopped by adding 100 µL of stop solution (sulfuric acid), and the optical density (OD) was measured at 450 nm using a microplate reader. The presence or absence of CHIKV-specific antibodies was determined by comparing the optical density (OD) values of the test samples to the assay-specific cut-off value calculated according to the manufacturer’s validated protocol. Samples with OD values equal to or above the cutoff were classified as positive, while those below were considered negative. According to the manufacturer’s validation data, the anti-CHIKV IgG ELISA assay demonstrates a reported diagnostic sensitivity of approximately 95% and a specificity exceeding 97% for the detection of Chikungunya virus–specific IgG antibodies. The assay cut-off value was calculated following the manufacturer’s protocol, based on the mean optical density (OD) of negative controls plus a defined constant. Samples with OD values equal to or greater than the calculated cut-off was interpreted as positive, while those below the cut-off were considered negative. The kit includes internal positive and negative controls to ensure assay validity and performance for each run. All testing procedures were conducted strictly according to the manufacturer’s instructions provided with the commercial ELISA kit, and standard quality control measures, including the use of supplied positive and negative controls, were applied to ensure assay accuracy, reproducibility, and biosafety. The anti-CHIKV IgG ELISA kit used in this study (Euroimmun, Lübeck, Germany) has been previously validated by the manufacturer and independent investigators. According to the manufacturer’s evaluation, the assay demonstrates a reported sensitivity of approximately 95–98% and a specificity of ≥98% for the detection of CHIKV IgG antibodies in human serum samples. Independent validation studies have similarly confirmed the high diagnostic accuracy of this assay, reporting sensitivities ranging from 93% to 99% and specificities above 97%, with minimal cross-reactivity against other arboviruses when used in non-endemic or low co-circulation settings. These performance characteristics support the suitability of the Euroimmun CHIKV IgG ELISA for population-based seroepidemiological investigations and retrospective exposure assessment. It is recognized that serological assays targeting alphaviruses may exhibit varying degrees of cross-reactivity due to shared antigenic epitopes within the family *Togaviridae*. However, the ELISA kit employed in this study utilizes recombinant CHIKV-specific antigens designed to minimize cross-reactivity with other alphaviruses. In addition, no other alphaviruses with known sustained human transmission have been documented in Iran, reducing the likelihood that detected IgG antibodies reflect exposure to non-CHIKV alphaviruses. Therefore, within the epidemiological context of the study area, IgG seropositivity was interpreted as indicative of prior exposure to Chikungunya virus. Confirmatory neutralization testing, such as plaque reduction neutralization tests (PRNT) or focus reduction neutralization tests (FRNT), was not performed in the present study. These assays require access to live Chikungunya virus and biosafety level-3 (BSL-3) laboratory facilities, which were not available during the study period. Consequently, the serological findings are based on ELISA-detected IgG antibodies and are interpreted as evidence of probable prior CHIKV exposure. The ELISA assay was used strictly as a qualitative diagnostic tool to classify samples as CHIKV IgG–positive or –negative according to the manufacturer’s cut-off criteria. No standard curve, quantitative IgG measurement, or linearity analysis was performed, and optical density values were not interpreted as antibody concentrations.

1. ***Data Analysis***

Data obtained from ELISA testing were entered into Microsoft Excel and subsequently analyzed using descriptive statistical methods. Each serum sample was classified as either positive or negative for Chikungunya virus (CHIKV) IgG antibodies based on the optical density (OD) cutoff values provided by the ELISA kit manufacturer. The frequency and percentage of positive and negative samples were calculated to determine the seroprevalence rate of CHIKV in the study population. The overall prevalence was computed by dividing the total number of CHIKV-positive samples by the total number of valid samples tested, and the result was expressed as a percentage. To provide a geographical perspective, the distribution of positive cases across the ten participating cities was also summarized. Findings were tabulated and visualized through appropriate tables and figures to support interpretation. No inferential statistical tests were conducted due to the descriptive nature of the study and the relatively limited sample size. However, the results serve as an important preliminary assessment of CHIKV circulation and provide a foundation for future, more extensive epidemiological investigations. To visualize the spatial pattern of seropositive cases, Figure 2 presents the geographic distribution of CHIKV IgG seroprevalence across Bushehr Province

1. ***Statistical Analysis***

Statistical analysis was performed using SPSS software (version 26.0). The primary objective was to determine the seroprevalence of anti-Chikungunya virus (CHIKV) IgG antibodies and to describe the distribution of positive cases across the ten surveyed cities in Bushehr Province. Descriptive statistics were used to summarize the data. The number and proportion of positive and negative samples were calculated, and the overall seroprevalence rate was expressed as a percentage. Additionally, city-wise frequencies of positive cases were reported to identify potential clustering or geographic variation in exposure to CHIKV. Due to the limited number of positive cases and the cross-sectional nature of the study, inferential statistical tests (e.g., chi-square tests or logistic regression) were not applied. The small sample size and low prevalence limited the statistical power for detecting significant associations between CHIKV positivity and demographic or geographic variables. Nevertheless, the descriptive results provide important insights for public health decision-making and serve as a baseline for hypothesis generation and future analytical studies on the epidemiology of CHIKV in Iran.

1. ***Reporting Standards***

This study was conducted and reported in accordance with the STROBE (Strengthening the Reporting of Observational Studies in Epidemiology) guidelines for cross-sectional studies. The STROBE checklist was followed to ensure transparency, completeness, and scientific rigor in the presentation of methods, results, and interpretations. All essential components such as study setting, participant eligibility criteria, sample size determination, data sources and measurement tools (including ELISA methodology), statistical approaches, and limitations have been clearly described. Ethical approval and informed consent procedures were also addressed in accordance with international standards for biomedical research involving human participants. Furthermore, all efforts were made to maintain internal consistency across sections and to minimize potential sources of bias through standardized data collection and laboratory testing protocols. The study’s adherence to established reporting standards enhances its credibility and facilitates its inclusion in future systematic reviews and meta-analyses.

1. ***Study Design***

This investigation employed a cross-sectional, seroepidemiological study design aimed at determining the prevalence of anti-Chikungunya virus (CHIKV) IgG antibodies among residents of Bushehr Province, Iran. The study was designed to capture a snapshot of CHIKV exposure within a defined population during a specific period, from July to September 2023. This timeframe was chosen to coincide with peak *Aedes* mosquito activity, driven by seasonal climatic conditions, which enhances the likelihood of virus transmission. The cross-sectional design was deemed appropriate for the early phase of epidemiological assessment in a region not previously characterized for CHIKV circulation. It provided an efficient means to estimate seroprevalence and detect evidence of prior infection based on immunoglobulin G (IgG) antibodies. These antibodies serve as markers of past exposure rather than active infection, making them suitable for identifying historical CHIKV exposure within the population.

The study was primarily exploratory and intended to lay the groundwork for future investigations. It serves as a foundation for potential longitudinal or case-control studies that could explore risk factors, temporal patterns, and the dynamics of CHIKV transmission. The study population consisted of voluntary adult participants (aged ≥18 years) who attended blood donation centers and hospitals across ten cities in Bushehr Province: Asaluyeh, Kangan, Bushehr, Dashtestan, Dashti, Deylam, Deyr, Ganaveh, Jam, and Tangestan (**Table 2**). The inclusion of multiple cities enhanced the representativeness of the sample, allowing for geographic comparisons of seroprevalence across different areas of the province. Eligibility criteria were carefully established to ensure the internal validity of the findings. Individuals presenting with acute febrile illnesses or those who had recently traveled to areas endemic for arboviruses were excluded from the study. This exclusion criterion was crucial in minimizing confounding by recent or imported infections, thereby ensuring that the antibodies detected in the study were indicative of autochthonous exposure within Bushehr Province. Standardized protocols were followed for all study procedures, including participant recruitment, blood sample collection, serum separation, storage, and ELISA testing. These protocols were designed to minimize inter-site variability and ensure methodological consistency across the different locations where samples were collected. Ethical considerations were strictly adhered to, and the study protocol received prior approval from the institutional review board (IR.SUMS.SCHEANUT.REC.1401.121). Written informed consent was obtained from all participants before their inclusion in the study.

This study's design, which focused on serological evidence of CHIKV exposure using the validated ELISA method, provided critical baseline data for public health surveillance and preparedness in Bushehr Province. Although molecular diagnostic methods such as RT-PCR are considered the gold standard for confirming acute CHIKV infections, this study relied on serological detection to assess past exposure within the general population. This approach was part of a phased surveillance strategy, serving as the first step in a broader research program that also includes ongoing molecular investigations of mosquito vectors and vertebrate hosts, as detailed in the doctoral thesis of the corresponding author. The absence of acute febrile cases during the sampling period, along with the limited window for detectable viremia (typically 5–7 days post-infection), further justified the reliance on serological data in this cross-sectional survey. The findings from this study, based on IgG antibodies, provide an important preliminary understanding of CHIKV exposure in Bushehr Province, which will inform future public health strategies and surveillance efforts.

1. ***Sample Size Determination and Participant Distribution***

The sample size for this cross-sectional seroepidemiological study was calculated based on standard statistical methodology for estimating prevalence in finite populations. In the absence of prior comprehensive seroprevalence data for Chikungunya virus (CHIKV) in Bushehr Province, an anticipated prevalence of 5% was conservatively assumed to ensure a sufficiently powered sample for initial surveillance purposes. The desired level of precision (margin of error) was set at 3%, and a 95% confidence level was adopted, corresponding to a standard normal deviate (Z) of 1.96.

The sample size (n) was estimated using the formula:


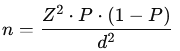


Where:

- Z=1.96Z = 1.96Z=1.96 (for 95% confidence)
- P=0.05P = 0.05P=0.05 (expected prevalence)
- d=0.03d = 0.03d=0.03 (precision)

Substituting the values yields:


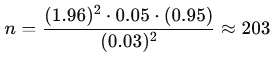


To accommodate anticipated issues such as participant attrition, refusal to consent, or sample quality failures, a total of 180 participants were ultimately enrolled due to practical constraints related to logistics, resource availability, and participant turnout. Although slightly below the calculated ideal sample size, the final cohort was deemed adequate for exploratory epidemiological purposes and provided a reliable estimate of CHIKV seroprevalence in the province.

The 180 participants were recruited from blood donation centers and hospitals distributed across ten cities in Bushehr Province, ensuring a broad geographic representation. The participant allocation was proportionally stratified based on the accessibility of healthcare facilities and blood centers in each city. This approach was intended to capture potential spatial variability in CHIKV exposure across diverse ecological and demographic settings.

The distribution of collected samples across the study sites was as follows:

- Bushehr City: 3 collection centers (total: 47 samples)
- Dashtestan: 3 centers (total: 45 samples)
- Dashti: 1 center (total: 28 samples)
- Asaluyeh and Kangan: 2 centers (total: 28 samples combined)
- Deylam, Deyr, Ganaveh, Jam, and Tangestan: 1 center each (total: 32 samples combined)

All blood samples were collected using standardized procedures, transported under temperature-controlled conditions, and processed uniformly for ELISA testing. This ensured consistency in specimen quality and minimized analytical variability across locations.

The resulting participant distribution reflects both the operational feasibility of sample collection and the intention to generate geographically informative seroprevalence data that can support regional risk assessment and guide vector surveillance strategies(Abbasi, 2025d; Abbasi, 2025f; Abbasi, 2025q; Abbasi, 2025s; Abbasi, 2026a; Abbasi, 2026b).

1. ***Study Variables***

This study investigated the seroprevalence of Chikungunya virus (CHIKV) through the detection of anti-CHIKV IgG antibodies using an ELISA-based serological method. To achieve this, several key variables both dependent and independent were identified and operationally defined to facilitate data collection, analysis, and interpretation (**Table 3**).

**Primary Outcome Variable**

- **Serological Status for CHIKV**: The primary dependent variable was the CHIKV IgG antibody status, determined through ELISA testing. This variable was dichotomously categorized as:
  - **Positive**: Optical density (OD) values at or above the manufacturer-defined threshold, indicating prior exposure to CHIKV.
  - **Negative**: OD values below the threshold, indicating no detectable past exposure.

**Demographic and Geographic Variables**

These variables were treated as independent or explanatory factors to contextualize the seroprevalence results:

- **City of Residence**: Recorded as a categorical variable representing one of the ten cities within Bushehr Province (e.g., Bushehr, Dashtestan, Dashti, etc.). This variable facilitated spatial analysis of potential differences in CHIKV exposure across urban and semi-urban settings.
- **Age Group**: Participants were categorized into predefined age brackets (e.g., 18–30, 31–45, 46–60, >60 years) to examine age-specific trends in seropositivity, although inferential analysis was not conducted due to limited sample size.
- **Sex**: Documented as male or female to assess any basic demographic imbalances in seroprevalence distribution.

**Eligibility Criteria Variables**

To ensure internal validity, the following inclusion-related variables were assessed:

- **Permanent Residency in Bushehr Province**: Coded as “yes” for eligible participants; this ensured that seropositivity would reflect local exposure.
- **Absence of Acute Febrile Illness at Time of Sampling**: Participants self-reported their current health status to exclude individuals with possible active arboviral infection.
- **Lack of Recent Travel to Endemic Areas**: Participants with recent travel (within the past three months) to known arbovirus-endemic regions were excluded to prevent confounding by imported cases.

**Laboratory Quality Control Variables**

- **Sample Adequacy**: Each serum sample was evaluated for volume and integrity. Samples failing to meet ELISA testing criteria were excluded from analysis.
- **Unique Identifier Code**: Each sample and participant were assigned a unique code to ensure traceability and maintain confidentiality while enabling linkage across data sources.

By clearly defining and categorizing these variables, the study ensured robust and systematic data collection and laid the foundation for transparent reporting and future multivariate analysis in expanded research settings.

1. ***Statistical Analysis and Generalizability***

***Statistical Analysis***

Data analysis in this study was primarily descriptive, in line with its cross-sectional and exploratory nature. All raw data including participant demographics and serological results were entered into Microsoft Excel and subsequently imported into SPSS software (version 26.0) for structured statistical evaluation. The primary analytical objective was to determine the seroprevalence of anti-Chikungunya virus (CHIKV) IgG antibodies in the study population. Each sample was classified as either positive or negative based on ELISA optical density (OD) readings compared against the manufacturer’s cutoff values. The overall prevalence was computed as the number of CHIKV-positive samples divided by the total number of valid tested samples, expressed as a percentage. Additionally, frequency distributions of positive and negative cases were calculated across different cities to provide a geographic perspective on potential clustering or variation in CHIKV exposure. Results were summarized in tabular and graphical formats (e.g., tables and bar charts) to support visual interpretation. Due to the limited number of seropositive cases (n = 5) and the modest sample size (N = 180), inferential statistical methods such as chi-square tests, logistic regression, or correlation analysis were not employed. Such analyses would lack statistical power and could yield unreliable or misleading conclusions. Instead, the findings are intended to serve as preliminary evidence to inform public health surveillance efforts and guide future studies with larger and more diverse populations.

***Generalizability***

While the study offers valuable insights into CHIKV exposure in Bushehr Province, several factors must be considered when interpreting the generalizability of the findings, The study included participants from ten cities, enhancing the regional representativeness of the data across urban and semi-urban areas within Bushehr Province (Geographic Coverage). Individuals were recruited from blood donation centers and hospitals, which may have introduced selection bias by favoring relatively healthy individuals who actively seek medical services. As such, the sample may not fully reflect more marginalized or rural populations with limited access to healthcare (Participant Selection). Data collection was conducted during a specific time frame (July to September 2023), corresponding with the active season for *Aedes* mosquitoes. The results, therefore, reflect a seasonal snapshot rather than year-round trends (Temporal Limitation). Only adults aged 18 years and older were included, excluding children and adolescents who may have different exposure risks or immune responses (Demographic Constraints). Despite these limitations, the findings provide a critical baseline estimate for CHIKV seroprevalence in a previously uncharacterized region. The data serve as an early warning signal for public health authorities and offer a foundation for longitudinal surveillance, targeted vector control strategies, and hypothesis-driven research across broader demographic and geographic cohorts.

1. ***Serological Testing and Rationale for Marker Selection***

***Serological Testing Approach***

The detection of past Chikungunya virus (CHIKV) infection in this study was conducted through the Enzyme-Linked Immunosorbent Assay (ELISA) technique, a widely recognized and validated method for seroepidemiological surveillance. The specific diagnostic tool utilized was a commercial ELISA kit designed for the qualitative detection of anti-CHIKV immunoglobulin G (IgG) antibodies in human serum or plasma samples. This assay type was selected due to its high specificity, operational simplicity, and suitability for large-scale screening under field conditions. Each test was conducted following the manufacturer’s standardized protocol. Serum samples were added to microplate wells pre-coated with CHIKV-specific antigens. Upon incubation, any existing CHIKV-specific IgG antibodies in the serum bound to the antigens. After a series of washing steps to remove unbound materials, an enzyme-linked anti-human IgG conjugate was added, followed by a substrate solution (TMB). A colorimetric change indicated the presence of specific antibodies, which was quantitatively measured by optical density (OD) at 450 nm. The results were interpreted using the cutoff values provided by the kit’s manufacturer, with OD readings at or above the cutoff considered positive, and those below deemed negative.

***Rationale for Marker Selection***

The study focused on IgG antibodies as the serological marker of choice for several key reasons, Unlike IgM antibodies, which are typically indicative of recent or acute infections, IgG antibodies persist for months or even years following initial infection. This makes IgG ideal for detecting prior CHIKV exposure in asymptomatic individuals or those with resolved infections, thus supporting population-level surveillance and risk assessment (Indicator of Past Exposure). Because Chikungunya virus (CHIKV) belongs to the genus *Alphavirus* (family *Togaviridae*), whereas dengue virus (DENV) and Zika virus belong to the genus *Flavivirus* (family *Flaviviridae*), significant antigenic cross-reactivity between CHIKV and flaviviruses is biologically unlikely. Therefore, the CHIKV IgG ELISA results in this study are unlikely to be confounded by prior dengue virus exposures, increasing diagnostic accuracy and specificity. This minimizes the risk of false positives in non-endemic or mixed-endemic settings (Reduced Cross-Reactivity). The use of an IgG-based ELISA allows for batch processing of samples, making it highly scalable and cost-effective for large seroprevalence studies. The logistical compatibility with centralized laboratory analysis, as well as its biosafety profile, further justified its selection (Feasibility in Field Settings). Since the study aimed to assess cumulative exposure and potential silent circulation of CHIKV in a non-endemic region, IgG antibodies served as the most appropriate marker. Detection of IgG supports the identification of areas with prior virus activity, even in the absence of clinically reported cases, and enables early warning for potential future outbreaks (Epidemiological Relevance). Overall, the choice of IgG ELISA testing provided a robust, ethically appropriate, and methodologically sound framework for evaluating seroprevalence in Bushehr Province. It facilitated the generation of actionable epidemiological data critical for public health planning, vector control prioritization, and outbreak preparedness.

1. ***Definition of DENV Positivity***

Although the primary focus of this study was on the seroprevalence of Chikungunya virus (CHIKV), the manuscript references broader arboviral surveillance efforts, including dengue virus (DENV) in regional contexts. As such, it is important to clarify the standard operational definition of DENV positivity, particularly in seroepidemiological studies employing enzyme-linked immunosorbent assay (ELISA) techniques.

***Serological Definition***

DENV positivity is typically determined based on the detection of anti-DENV IgG or IgM antibodies in serum samples using commercial ELISA kits, indicates recent or acute DENV infection, generally appearing within the first week after symptom onset and persisting for several weeks (IgM Positivity). Suggests past exposure to DENV and may persist for years, reflecting long-term immune memory (IgG Positivity). In ELISA-based testing, a sample is classified as positive for DENV when the optical density (OD) exceeds the threshold (cutoff) value defined by the kit manufacturer. These thresholds are often derived from internal controls and population-based reference ranges.

***Cross-Reactivity Considerations***

It is crucial to note that cross-reactivity between flaviviruses particularly DENV, Zika virus, and other related arboviruses is a recognized challenge in serological testing. Therefore, DENV positivity via ELISA should ideally be confirmed by more specific assays, such as plaque reduction neutralization tests (PRNT), when distinguishing between flavivirus infections is necessary, particularly in endemic or co-endemic settings. In the context of the current study, while DENV positivity was not directly assessed, the distinction is relevant for future integrated surveillance protocols that aim to differentiate between multiple arboviruses circulating within a region. Proper serological interpretation, with consideration for cross-reactivity and timing of infection, is essential for accurate disease burden estimation and guiding public health responses.
